# Antiosteoporotic Activity of Genistein Aglycone in Postmenopausal Women: Evidence from a Post-Hoc Analysis of a Multicenter Randomized Controlled Trial

**DOI:** 10.3390/nu9020179

**Published:** 2017-02-22

**Authors:** Vincenzo Arcoraci, Marco Atteritano, Francesco Squadrito, Rosario D’Anna, Herbert Marini, Domenico Santoro, Letteria Minutoli, Sonia Messina, Domenica Altavilla, Alessandra Bitto

**Affiliations:** 1 Department of Clinical and Experimental Medicine, University of Messina, 98100 Messina, Italy; varcoraci@unime.it (V.A.); fsquadrito@unime.it (F.S.); hrmarini@unime.it (H.M.); santisi@hotmail.com (D.S.); lminutoli@unime.it (L.M.); abitto@unime.it (A.B.); 2 Department of Neurosciences and Nemo Sud Clinical Center, University of Messina, 98100 Messina, Italy; rdannna@unime.it (R.D.); smessina@unime.it (S.M.); 3 Department of Paediatric, Gynaecological, Microbiological and Biomedical Sciences, University of Messina, 98100 Messina, Italy; daltavilla@unime.it

**Keywords:** bone mineral density, genistein, postemenopausal osteoporosis

## Abstract

Genistein has a preventive role against bone mass loss during menopause. However, experimental data in animal models of osteoporosis suggest an anti-osteoporotic potential for this isoflavone. We performed a post-hoc analysis of a previously published trial investigating the effects of genistein in postmenopausal women with low bone mineral density. The parent study was a randomized, double-blind, placebo-controlled trial involving postmenopausal women with a femoral neck (FN) density <0.795 g/cm^2^. A cohort of the enrolled women was, in fact, identified at the baseline as osteoporotic (*n* = 121) on the basis of their T-score and analyzed thereafter for the 24 months’ treatment with either 1000 mg of calcium and 800 IU vitamin D3 (placebo; *n* = 59); or calcium, vitamin D3, and Genistein aglycone (54 mg/day; genistein; *n* = 62). According to the femoral neck T-scores, 31.3% of the genistein and 30.9% of the placebo recipients were osteoporotic at baseline. In the placebo and genistein groups, the 10-year hip fracture probability risk assessed by Fracture Risk Assessment tool (FRAX) was 4.1 ± 1.9 (SD) and 4.2 ± 2.1 (SD), respectively. Mean bone mineral density (BMD) at the femoral neck increased from 0.62 g/cm^2^ at baseline to 0.68 g/cm^2^ at 1 year and 0.70 g/cm^2^ at 2 years in genistein recipients, and decreased from 0.61 g/cm^2^ at baseline to 0.60 g/cm^2^ at 1 year and 0.57 g/cm^2^ at 2 years in placebo recipients. At the end of the study only 18 postmenopausal women had osteoporosis in the genistein group with a prevalence of 12%, whereas in the placebo group the number of postmenopausal women with osteoporosis was unchanged, after 24 months. This post-hoc analysis is a proof-of concept study suggesting that genistein may be useful not only in postmenopausal osteopenia but also in osteoporosis. However, this proof-of concept study needs to be confirmed by a large, well designed, and appropriately focused randomized clinical trial in a population at high risk of fractures.

## 1. Introduction

Estrogen deficiency is a major cause of osteoporosis worldwide. Hormone Replacement Therapy (HRT) increases the bone mineral density (BMD) and decreases the fracture risk, but the adverse effects limit its use in a large cohort of subjects [1,2,3]. In the last few years, many researchers have focused their attention on natural alternatives to HRT, such as phytoestrogen. Epidemiological data indicate that women consuming high amounts of phytoestrogens, in the form of soy-derived dietary products, have less menopausal symptoms than those on Western diets, and consequently, it was assumed that bone mineral density may be favorably influenced by phytoestrogens [4,5]. A major phytoestrogen is genistein, which prevents bone mass loss and improves quality of life without the harmful estrogenic activity on reproductive tissues, or genotoxic effects in postmenopausal women, at least at the dose of 54 mg/day [6,7,8,9,10,11,12]. We have studied the effects of pure genistein (in its aglycone form) on BMD and bone metabolism in a large cohort of postmenopausal women [6], showing that a long-term intake of genistein produced a positive effect on bone metabolism with no clinically significant adverse effects on the breast and uterus [7,8,9]. Overall, these results agree with previous intervention trials in which >40 mg/day of genistein equivalents yielded the most positive effects on bone mineral density and bone markers when compared to other trials with lower amounts of genistein [13,14,15,16]. Animal studies suggest that genistein has an antiosteoporotic activity, improving bone morphology parameters in osteoporotic ovarictomized mice [17] and preventing bone fragility in rats with glucocorticoid-induced osteoporosis [18]. To date, no data are available on osteoporotic subjects, however a re-analysis of the 389 postmenopausal women enrolled in our previous trial, evaluating the efficacy of genistein in osteopenic women, revealed that a significant number was actually osteoporotic, according to the T-score. The aim of this study was to perform a post-hoc analysis to evaluate the efficacy of genistein aglycone on bone mineral density in women with osteoporosis.

## 2. Materials and Methods

### 2.1. Design Overview, Setting, Participants

A post-hoc analysis was carried out using data from the main multicenter randomized controlled trial “Effects of the phytoestrogen genistein on bone metabolism in osteopenic postmenopausal women”. The study subjects and methods are described in detail elsewhere [6,7,8]. The protocol is consistent with the principles of the Declaration of Helsinki, was approved by the Ethics Committee of Palermo University (RODA-12254) and the participants gave their written informed consent. The trial included 389 postmenopausal women, randomly assigned to receive the isoflavone genistein (*n* = 198), or placebo (*n* = 191). Placebo and genistein (54 mg/day) tablets were identical in appearance and taste. The purity of genistein was >98%. All patients were co-prescribed with 1000 mg calcium carbonate and 800 IU vitamin D3, in two tablets. The bone mineral density was assumed as the primary outcome. The femoral neck (FN), lumbar spine (LN), and total hip (TH) bone mineral density, were measured by Dual-Energy X-ray Absorptiometry (DXA; Hologic QDR 4500 W, Technologic Srl, Turin, Italy) at baseline and after 12 and 24 months of treatment as previously published [6,7,8]. In addition, the ten-year fracture probability was assessed with the Fracture Risk Assessment (FRAX) tool, as a secondary outcome.

### 2.2. Study Population

This primary analysis included all postmenopausal women with osteoporosis at the femoral neck. A cohort of the enrolled postmenopausal women was identified at the baseline as osteoporotic (*n* = 121) on the basis of their T-score on the femoral neck and were analyzed thereafter for the 24 months’ treatment with either placebo (*n* = 59) or genistein (*n* = 62). Data from postmenopausal women with osteopenia are also shown to assess whether these patients had similar beneficial effects on BMD and incidence of adverse events. 

### 2.3. Assessment of Fracture Risk

The ten-year fracture probability was assessed with the FRAX tool (version 2.0; University of Sheffield, South Yorkshire, England) in osteoporotic and osteopenic patients. FRAX is a computer-based algorithm [19] that provides models for the assessment of fracture probability in men and women. In this analysis, FN BMD was included since fracture risk prediction is enhanced with the input of BMD.

The probability of fracture is calculated in women according to age, body mass index, and dichotomized risk variables that comprise: a prior fragility fracture, parental history of hip fracture, current tobacco smoking, ever long-term use of oral glucocorticoids, rheumatoid arthritis, other causes of secondary osteoporosis, and alcohol consumption of three or more units daily [20]. 

### 2.4. Statistical Analysis

Descriptive statistical analyses were performed to evaluate basal demographic and clinical characteristics. All results were expressed as mean with standard deviation (SD) for continuous variables, and absolute and percentage frequencies for categorical variables. All variables were evaluated at basal time and after 12 and 24 months of treatment and absolute values were evaluated in both genistein and placebo patients to verify differences between the groups. The primary efficacy data on BMD were analyzed as already described [6,7,8]. The significance of differences in mean values between placebo and genistein groups at year 1 and 2 was assessed by two-way analysis of variance (2-way ANOVA). A *p* value of 0.05 or less was considered statistically significant and 95% confidence intervals were calculated wherever possible. Statistical analysis was performed by using SAS software, version 9.1 (SAS Institute, Inc., Cary, NC, USA).

## 3. Results

Of the 389 postmenopausal women who were enrolled in the main study, 121 (31.1%) were osteoporotic and were included in this analysis. The baseline characteristics of the osteoporotic and osteopenic women are shown in Table 1. The number of osteoporotic women was similar in both groups: 59 (30.9%) in the placebo group and 62 (31.3%) in the genistein group. 

**Figure 1:**
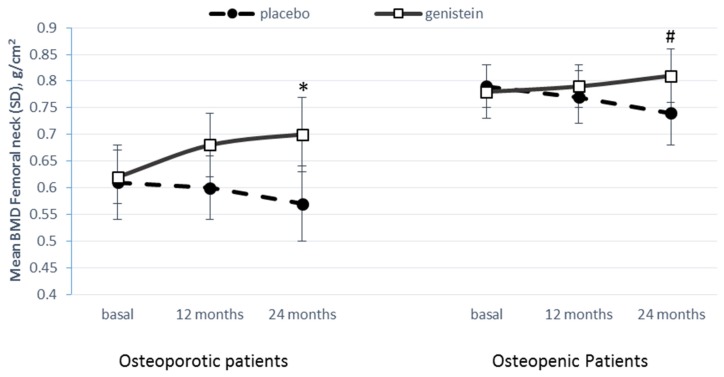
Femoral Neck Bone mineral density changes in absolute values over time in placebo and genistein group. (**left**) Femoral neck Bone Mineral Density changes in osteoporotic postmenopausal women groups; (**right**) Femoral neck Bone Mineral Density changes in osteopenic postmenopausal women groups. 2-way ANOVA: Over time, genistein vs. placebo: * Treatment *p* = 0.0046; # Treatment *p* = 0.0130; * Time *p* = 0.0068; # Time *p* = 0.4929; * Interaction *p* = 0.0073; # Interaction *p* = 0.0241.

Mean bone mineral density (BMD) at the femoral neck increased from 0.62 g/cm^2^ ± 0.05 (SD) at baseline to 0.68 g/cm^2^ ± 0.06 (SD) at 1 year and to 0.70 g/cm^2^ ± 0.07 (SD) at 2 years in the genistein recipients. Conversely, in the placebo group, BMD decreased from 0.61 g/cm^2^ ± 0.07 (SD) at baseline to 0.60 g/cm^2^ ± 0.06 (SD) at 1 year and to 0.57 g/cm^2^ ± 0.07 (SD) at 2 years. Over time the BMD significantly improved in the genistein group compared to the placebo group (*p* = 0.0046). Moreover, a significant time effect (*p* = 0.0068) and treatment-time interaction (*p* = 0.0073) were also observed. Similar trends, on bone mineral density at the femoral neck, were observed in osteopenic patients when considering treatment (*p* = 0.0130) and treatment-time interaction (*p* = 0.0241), while no significance was found for the time effect (*p* = 0.4929) (Figure 1). In genistein-treated osteoporotic patients, a significant increase of BMD at the lumbar spine (from 0.82 ± 0.08 at baseline to 0.85 ± 0.09 at 1 year and to 0.88 ± 0.08 at 2 years) and total hip (from 0.73 ± 0.06 at baseline to 0.80 ± 0.07 at 1 year and to 0.82 ± 0.08 at 2 years) was observed.

Treatment also affected the bone turnover rate. Indeed, in genistein-treated osteoporotic women, B-ALP increased from 10.0 ± 2.2 at baseline to 13.8 ± 2.3 after 1 year and to 14.6 ± 2.2 at 2 years, while no differences were observed in the placebo group (basal: 10.8 ± 1.8; 1 year 10.3 ± 1.6; 2 year 10.4 ± 1.6).

Over time a significant treatment effect (*p* < 0.01), time effect (*p* < 0.01), and treatment-time interaction (*p* < 0.01) were observed using the 2-way ANOVA. Similar results were observed in osteopenic women. D-Pyr excretion in osteoporotic patients was affected only by time (*p* = 0.002).

According to the World Health Organization (WHO) classification criteria, at baseline, the number of postmenopausal women with osteoporosis was 62 in the genistein group and 59 in the placebo group. At the end of the study only 18 (29%) genistein-treated subjects were osteoporotic, while no changes were observed in the placebo group. 

Gastrointestinal side effects were the most common reason for dropout, possibly because of the presence of calcium. There were no significant changes in routine biochemistry, liver function, or hematology results. The daily administration of 54 mg of genistein did not cause any significant change in the endometrial thickness.

## 4. Discussion

In a cohort of postmenopausal osteoporotic subjects, we confirmed the protective effects of genistein treatment on bone loss, as previously observed in osteopenic women. Postmenopausal osteoporosis significantly increases the risk of fractures and requires adequate prevention and appropriate medical treatment [21]. Since the lack of estrogen is the main etiopathogenetic element of postmenopausal bone loss, as a result, estrogen replacement therapy represents an established treatment for the prevention of osteoporosis [22]. However, the possibility of side-effects related to the estrogenic treatment requires therapeutic alternatives [23].

We previously showed that a daily administration of genistein aglycone produced a net gain in bone mass after 2 years of therapy in postmenopausal women with low bone mineral density and low fracture risk [6,7]. Although BMD and the bone markers previously assessed in our clinical trial are considered good surrogates of bone strength and bone quality, they may not correlate perfectly with a reduction in fracture risk. Fracture risk can be assessed more accurately by considering more variables than BMD alone, by using the FRAX tool. In this post-hoc analysis, our population had a medium-high fracture risk according to the FRAX scores; in fact, at baseline a 10-year fracture probability at the femur was 4.1 (1.9) in the placebo group and 4.2 (2.1) in the genistein group. This post-hoc comparison was made between the two treatment groups, showing for the first time that genistein aglycone (54 mg/day) plus calcium and vitamin D3 compared to only calcium and vitamin D3 supplementation significantly reduced the rate of osteoporosis, increasing BMD in postmenopausal women with medium-high 10-year fracture risk at the femur site. This analysis shows novel and additional findings distinct from those previously published [6,7,8]. More specifically, it appears that genistein has possible implications for the reduction of fracture risk in postmenopausal women with osteoporosis. 

We believe that these data are particularly intriguing: in fact, this surrogate result on fractures overcomes one of the main limitations of our previous study, which was the lack of data on this major outcome. Additionally, the possibility to include, through the FRAX calculation, different risk factors in every patient enrolled in our clinical trial represents a strong advantage in terms of analysis for therapeutic efficacy.

Women enrolled in the parent trial were osteopenic rather than osteoporotic and this might represent a limitation for the current analysis; nevertheless, our study [6] remains, to date, the largest double-blind, placebo-controlled trial on genistein aglycone in the literature. To fill this gap, additional trials involving osteoporotic women are needed to further confirm the present results.

## 5. Conclusions 

In conclusion, genistein plus calcium and vitamin D3 treatment demonstrated similar effects in terms of BMD increase at the femur versus placebo over 2 years in the subgroup of patients with osteoporosis. Collectively, these data confirm the positive and unique role of genistein aglycone, suggesting that it may be the most active isoflavone for treating postmenopausal bone loss, with a time-dependent effect, and suggesting that a long-term intake of genistein produces ongoing effects on bone health. This post-hoc analysis represents a proof-of concept study: it points out that genistein may counteract osteoporosis in postmenopausal women. However, this proof-of concept study needs to be confirmed by a large, well designed, and appropriately focused randomized clinical trial in a population at high risk of fractures. Currently, a clinical trial is investigating the effects of genistein on the fracture rate in glucocorticoid-induced osteoporosis and the final results are expected to be released in late 2018. These results will definitely clarify whether genistein may be useful not only in osteopenia but also in osteoporosis.

## Figures and Tables

**Table 1:** Baseline characteristics of postmenopausal women with osteoporosis and osteopenia in both groups.

Variable	Osteoporotic		Osteopenic	
	Placebo (59)	Genistein (62)	Placebo (132)	Genistein (136)
Mean age (SD), year	54.3 (2.4)	54.5 (2.9)	54.6 (2.7)	54.8 (2.2)
Mean body mass index (SD), Kg/m^2^	25.2 (3.0)	25.5 (2.8)	25.4 (3.9)	24.8 (3.7)
Mean time since menopause (SD), m	69.4 (44.7)	68.3 (39.2)	69.1 (35.9)	66.4 (38.3)
Mean BMD Femoral neck (SD), g/cm^2^	0.61 (0.07)	0.62 (0.05)	0.79 (0.04)	0.78 (0.05)
Mean BMD Lumbar Spine (SD), g/cm^2^	0.81 (0.10)	0.82 (0.08)	0.85 (0.10	0.85 (0.08)
Mean BMD Total hip (SD), g/cm^2^	0.72 (0.08)	0.73 (0.06)	0.93 (0.04)	0.92 (0.05)
B-ALP (μg/L)	10.8 (1.79)	10.0 (2.21)	10.0 (1.87)	10.6 (2.09)
D-Pyr (pmol/μmol of urinary creatinine)	22.0 (6.92)	22.7 (7.86)	20.6 (5.19)	21.2 (3.97)
**FRAX index**				
Major fractures (SD)	6.5 (2.8)	6.6 (2.4)	3.3 (0.5)	3.4 (0.5)
Femur fractures (SD)	4.1 (1.9)	4.2 (2.1)	0.7 (0.1)	0.7 (0.1)

BMD: Mean bone mineral density.
